# A novel high-resolution test battery for language mapping in awake craniotomy: preliminary perioperative results in neurooncologic patients

**DOI:** 10.3389/fonc.2026.1762147

**Published:** 2026-05-18

**Authors:** Marion Rapp, Peter Pieperhoff, I-An Tan, Katrin Amunts, Michael Sabel, Yosef Grodzinsky

**Affiliations:** 1 Department of Neurosurgery, University Hospital Duesseldorf, Duesseldorf, Germany; 2 Brain Cancer Center, Beta Clinic Bonn, Bonn, Germany; 3 Forschungszentrum Jülich, Institute of Neuroscience and Medicine (INM-1), Jülich, Germany; 4 Edmond and Lily Safra Center for Brain Research, The Hebrew University, Jerusalem, Israel; 5 C. & O. Vogt Institute for Brain Research, University Hospital Duesseldorf, Heinrich Heine University Duesseldorf, Duesseldorf, Germany

**Keywords:** awake surgery, brain tumor, glioma, language testing, neurooncology

## Abstract

**Objective:**

Recent data underscore the impact of supramarginal resection of gliomas on prolonging survival. The state-of-the-art approach, aimed at preventing permanent postoperative neurological deficits, involves intraoperative functional mapping and monitoring of eloquent regions. Awake surgery has thus become the gold standard method to assess language function. However, at present, no test battery is sensitive enough to reliably detect and map complex language functions intraoperatively–functions that are crucial for patients’ communicative abilities and strongly impact their quality of life. Except for the dated (60 years old) sentence-level Token Test, patients’ language is typically examined at the word level (e.g., picture naming and identification). We have developed and administered six highly sensitive sentence-level tests that reflect a broad range of supra-lexical language functionalities. To assess test sensitivity, we compared the results of our 6 new tests to those of a battery of 11 standard tests.

**Patients and methods:**

In this retrospective, monocentric analysis, 48 patients with eloquently located tumors and electively planned awake surgery were analyzed pre- and postoperatively using standard language assessment and our app-based high-resolution test battery.

**Results:**

A novel set of supra-lexical simple and complex sentence-level tests is at the heart of this work. Most patients performed equally well on simple sentence-level and standard tests. Performance on linguistically complex sentence-level tests was significantly lower and showed high between-patient variability, as was demonstrated by a variety of statistical analyses.

**Conclusion:**

Our results underscore the value of tests that feature complex sentence-level stimuli used in everyday communication. The more complex tests exhibit significantly higher sensitivity compared to standard language assessment. Our results thus demonstrate the utility of such tests in preserving patients’ postoperative quality of life and call for their widespread use in awake surgery.

## Introduction

If left untreated, gliomas of all grades inevitably lead to catastrophic neurological deficits and death. At present, gliomas are treated surgically, with the goal of maximizing life expectancy (LE) (1) while preserving quality of life (QoL), which often requires awake surgery during which the patient’s cognitive functions are repeatedly tested (2).

Looking back, we note that for decades, glioma therapy was mostly palliative, with only a minor positive impact on patients’ LE. Chemo- and radiotherapies were later added, leading to little LE improvement. Surgical procedures were limited, as surgery often resulted in additional functional impairment that degraded patients’ QoL. Yet more recently, prospective randomized trials demonstrated the impressive impact of gross total resection (GTR) on LE (3), and the possibility of surgery in high-grade gliomas was reconsidered. Moreover, several groups developed a radical supramarginal resection approach and showed that it brought about near-normal life expectancy for low-grade gliomas (4–7) and an almost threefold effect on LE for high-grade gliomas (8). Surgical procedures thus made a comeback, with the focus shifting from pure lesionectomy (3) to supramaximal resection incorporating MR FLAIR/T2 imaging-positive tumor areas (6). Yet this advancement may put QoL at risk: as the principle of supramarginal resection is defined by intentionally resecting into the functional infiltration zone, a permanent neurological deficit may result, which in turn threatens to severely reduce the potential survival and QoL gains (6, 9).

This neurooncosurgical dilemma concerns the “onco-functional balance” (10): how to weigh the extent of LE-elevating resection against the need to preserve neurological and cognitive functionality for the sake of adequate QoL. This dilemma forces dramatic neurosurgical decisions that must be made both pre- and intraoperatively. This crucial decision-making process is most obvious when the preservation of language functions is at issue because language is the main means of human expression, communication, and socializing. Modern neurosurgery has made extensive efforts to preserve functionality through awake surgery that allows for the mapping of language and communicative functions intraoperatively.

Therefore, performing supramaximal resection while securing QoL depends on tools that allow precise identification of functional anatomy.

Similar to other intraoperative functional mapping approaches, the anatomical structures that support language functions are detected in awake surgery via monopolar and bipolar stimulation during language testing on both the cortical and the subcortical levels (11). Intraoperative language testing in the awake setting is based on our understanding of the language system as a set of eloquent cortical areas and subcortical fiber tracts, each entrusted with some specific function(s) (12, 13). How do we choose the right test? We are informed by a large body of results from neuropsychological and neurocognitive investigations of the human brain in health (mostly through functional imaging) and in brain disease that affects language (mostly through stroke-induced aphasia) (14–17).

These have helped to build a picture that we seek to incorporate directly into our clinical protocol in order to localize language mechanisms in the individual patient by identifying eloquent and silent areas in the vicinity of the area to be resected. As linguistic functions are found in multiple loci but not everywhere in the brain, test choices should depend on tumor localization (12). Crucially, the soundness of this reasoning and its relevance to patients’ QoL depend on the breadth of our language test battery and on its neurological relevance. We want to test multiple functions, but only those that are supported by the implicated brain areas.

Unfortunately, most neurooncological centers presently use a rather restricted set of tests, limited to the word level. Few studies have focused on comprehension abilities (with some exceptions, to which we later return) (18–21), and even fewer have probed supra-lexical (i.e., regularities that are at a level above the single word, namely, phrases and sentences, which require complex testing) levels during awake neurooncological surgery, despite the fact that these are critical for patients’ daily communication: it is typically conducted not through words, but via systematically selected sentences that humans construct and analyze in every speech act. The rich array of language-related tasks we perform daily enables us to make and interpret statements, to ask and answer questions, to evaluate the truthfulness of linguistic statements in context, and to draw conclusions that lead to actions. These abilities–critical for QoL–are often left untested on the operating room (OR).

To fill this lacuna, we have developed an app-based language test comprising six novel comprehension tests beyond the word level, namely, sentences of different types and complexity levels. All tests were selected on the basis of well-established results from cognitive neuroscience in health and in focal brain disease.

Below, we briefly describe initial pre- and postoperative results of these tests in comparison to standardly used language tests13, the rationale behind them, their implementation on an iPad app we developed for administration, and the outcome. We conclude by presenting a longitudinal study of a single case to provide evidence for the value of the high-resolution Jülich-Brain Atlas11 in matching functional results with microanatomical maps.

## Methods

### Study design

We performed a monocentric, retrospective data analysis of pre- and postoperative language assessments of glioma patients with eloquently located tumors who were electively admitted to our hospital for awake craniotomy. Patients received a battery of 11 standard tests and 6 novel tests, both pre- and postoperatively. The study was approved by the ethics committee of the medical faculty at Heinrich-Heine University Düsseldorf (Ethic ID: 2021-1802; 2020-1042).

### Patient selection criteria

Inclusion criteria for this data analysis were: (1) planned awake craniotomy due to a brain tumor near eloquent anatomical structures, (2) complete pre- and postoperative dataset of standard language tests, and (3) complete pre- and postoperative dataset of the new, app-based language assessment.

### Standard language assessment

In keeping with a widespread tradition of OR-testing, we used 11 standard tests: (1) picture naming, (2) picture identification, (3) weekdays, (4) months, (5) the standard Stroop test, (6) famous personalities, (7) the Pyramids and Palm Trees Test (PPTT)–grouping of meaning-related words, (8) counting, (9) calculation, (10) reading, and (11) the Token Test (12, 14, 18). The first seven tests are at the single-word level; the next two concern basic arithmetic; finally, the last two tests, the Token Test (sentence comprehension) and a reading test (reading a paragraph aloud), involve a small set of sentence stimuli. These types were grouped into a single group: standard tests.

### Supra-lexical tests – simple and complex

#### App-based extended language assessment

We devised a new, (neuro)linguistic-evidence-based sentence-level test battery that currently consists of three types of linguistically simple (LING SPL) and three types of linguistically complex (LING CPX) sentence comprehension tests (six subsets in total) (22–25), where sentence complexity is formally defined and the choices are based on neuropsychological and neuroimaging evidence (22–26). The task is a Sentence-to-Picture Matching (SPM) binary forced-choice task: an auditory sentence, simultaneously displayed on an 11-inch iPad screen, is juxtaposed with two picture buttons. The patient is asked to press one button (**Table 1**). The sentence types were all in German, with examples listed in **Table 1**, as follows:

**Table 1:** Language stimuli and the images that accompany them.

Condition	Sentence type	Image(s)
1. REQuest	Simple (LING-SPL): The woman who is pushing the girl is blond	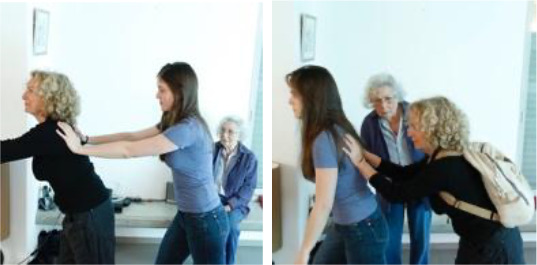 (16 token stimuli)
	Complex (LING-CPX): The woman who the girl is pushing is blond	
2. Question	Simple (LING-SPL): Which man is combing the girl?	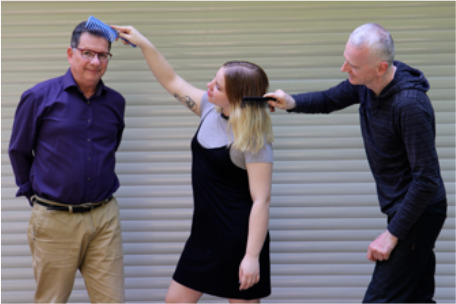 (12 token stimuli)
	Complex (LING-CPX): Which man is the girl combing?	
3. COMParison	Simple (LING-SPL): In the picture there are *more* blue than yellow dots	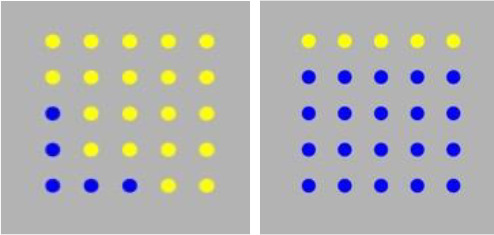 (16 token stimuli)
	Complex (LING-CPX): In the picture there are *fewer* yellow than blue dots

1. Syntactically simple and complex requests, implemented in relative clauses; participants were asked to select the matching picture, where the foil depicted a reversal of roles.
2. Syntactically simple and complex German questions; participants were asked to answer a question by pressing the correct character (out of two).
3. Semantically simple and complex comparative sentences; the foil depicted a reversed relation between two quantities.

Each sentence type has a number of different tokens. The six conditions constitute the independent (manipulated) variable; the dependent (measured) variable is the error rate–the proportion of correct responses in our binary choice task. Importantly, the structure of the three tests is fully counterbalanced, each featuring a simple and a complex version, with the exact same number of words and syllables. This property of the battery means that experience gained through repeated testing, such as in our study protocol (pre-, intra-, and post-surgical testing), gives participants no advantage and is not expected to reduce errors. Moreover, the error rate cannot be explained by reference to simple variables such as the number of words or their frequency of occurrence (27, 28). The different stimulus types were clustered into linguistically simple (LING SPL) and linguistically complex (LING CPX) stimulus sets.

Zero-error performance has been verified with neurologically intact individuals for all tests used and is therefore taken as a baseline.

### Statistical analyses

**Figure 1:**
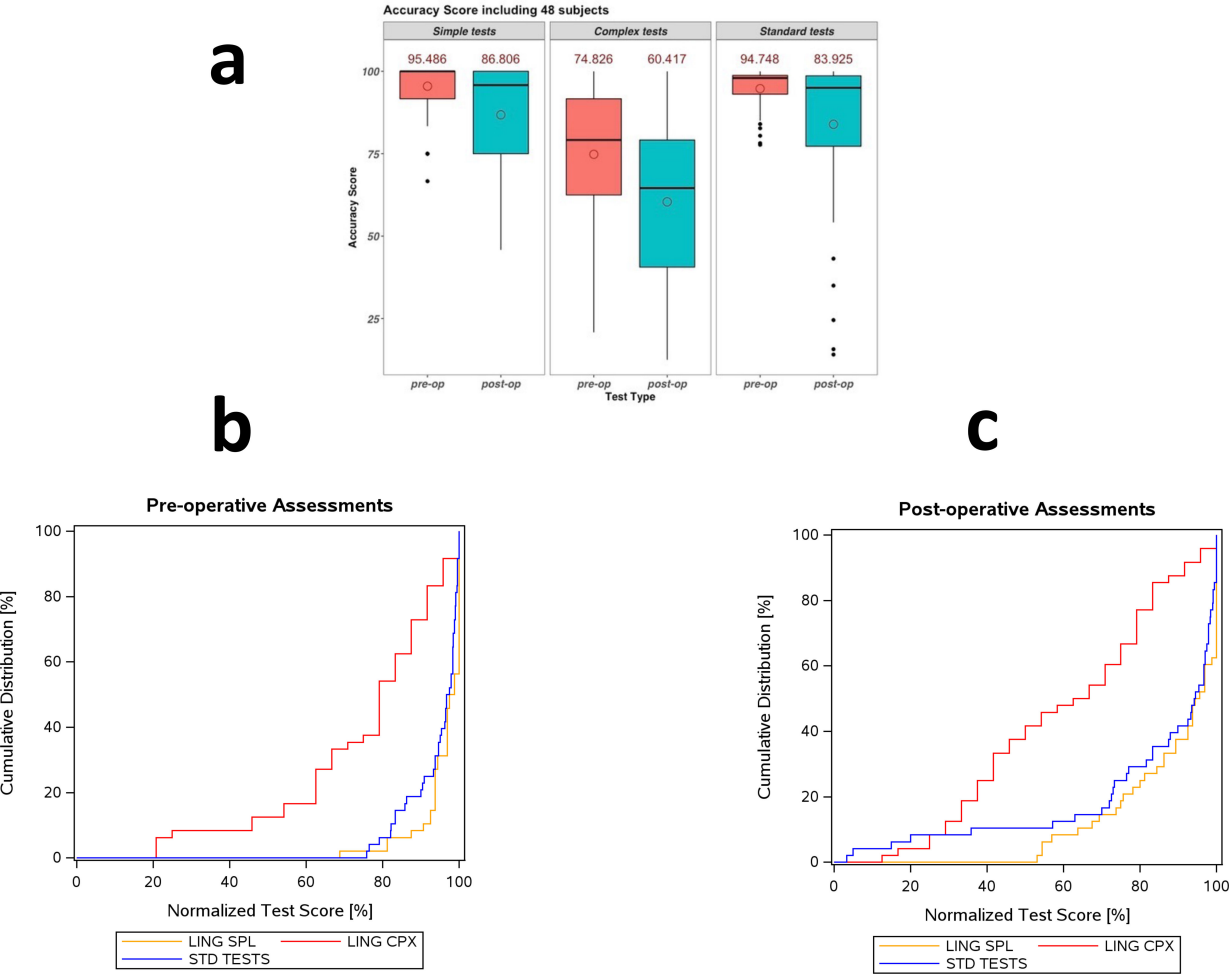
Inter-test-type comparisons (Standard, LING SPL, and LING CPX). **(a)** Mean accuracy scores per test type (LIN_SPL, LING_CPX, Standard). **(b)** Cumulative distribution plot (normalized % correct, x-axis) for the three pre-operative test types (normalized number of patients, y-axis): LING SPL (orange), LING CPX (red), and Standard (blue). The LING SPL tests yielded the lowest error rate, (though comparable to the Standard tests), whereas LING CPX yielded the highest error rate, as indicated by the leftward shift. **(c)** same results for the postoperative test types **(b)**, for the postoperative test types.

Individual mean values of each test group (LING SPL, LING CPX, Standard) and time point (pre-, postoperative) were calculated (**Figure 1**), e.g., the mean value of LING-CPX tests (t) of subject (i) (*f_t_ (i*, τ) = test score of subject *i* in test *t* [belonging to LING-CPX] at time-point τ):


$$m_{LING-CPX,\tau }(i)=\frac{1}{3}\sum_{t\in LING-CPX}f_{t,\tau }(i)$$

The corresponding sample mean value is the average over all subjects:


$$\mu_{LING-CPX,preop}=\frac{1}{N}\sum_{i=1}^{N}m_{LING-CPX,preop}(i)$$

The significance of differences between these sample mean values was examined by a non-parametric permutation test with 10,000 iterations, separately for each time point (i.e., *µ_LING-CPX,preop_*(*i*) vs. *µ_Standard,preop_*(*i*), etc.). Next, individual changes of these test-group mean values between the pre- and post-operative time points were calculated,


$$\Delta m_{LING-CPX}(i)=m_{LING-CPX,postop}(i)-m_{LING-CPX,preop}(i)$$

and the significance of differences between test groups was examined as before, using a permutation test. Finally, in order to exploratorily analyze the impact of each single test on these test-type related results, differences between the sample mean values of single tests were examined in the same way. i.e. every test of LING-SPL vs. every test of LING-CPX vs. every Standard-test, yielding 3 × 3 + 3 × 11 + 3 × 11 = 75 tests. Significance level in most of these tests is exceedingly high (see **Supplemental Table 1**), and would persist under any test that corrects for multiple comparisons.

To detect significant differences between the mean values of tests pre- and postoperatively, we therefore used a non-parametric permutation test (with 10^5^ iterations).Interactions between test type and time of testing (pre/post-OP) were analyzed by permuting differences between scores. The permutation tests provide a perspective based on group means.

The Kolmogorov-Smirnov test was applied with the aim of determining whether the group results on each test type originated from the same distribution. The scores achieved by subjects in the three different test types (LING-SPL, LING-CPX, and Standard) in the pre- and postoperative (post-OP) assessments were analyzed to detect differences in their cumulative distributions: Whereas a linear increase in the cumulative distribution would be desirable because it indicates that the test scores are evenly distributed, an e.g. at the upper end concentrated distribution would indicate the occurrence of ceiling effects in the tests. Therefore, the cumulative distributions of the normalized scores (*x*) of each test (*j*) and time-point were plotted, using the formula:


$$F_{f}(x)=\frac{\#\{x_{i}\leq x\}}{N}\cdot 100$$

(*N* = number of subjects, *x*_1_, *x*_2_,…,*x_N_* = scores in a given test, #{xi ≤ x}= the count of data points in a dataset that are less than or equal to a specific value x) as an estimate of the distribution function.

The significance of differences between the distribution functions of the test types were examined by applying the Kolmogorov-Smirnov (KS) test between each pair of test types. The asymptotic KS statistic was calculated. *x*_1_, *x*_2_,…,*x_n_p__*.


$$KS_{a}=\underset{i}{\max}|F_{1}(x_{i})-F_{2}(x_{i})|\frac{\sqrt{n_{p}}}{2}$$

The NPAR1WAY procedure of the software SAS^®^ 9.4 (SAS Institute Inc., Cary, USA) was used for these computations.

## Results

### Patient cohort

From August 2020 to August 2023, 464 neurooncological patients were electively admitted to our department for awake craniotomy for malignant brain tumors and underwent pre- and postoperative behavioral testing. Of these, we included only patients for whom we had a complete set of test scores (for all 17 tests), administered both pre- and post-OP. To obtain reliable results, we enforced a strict protocol requiring the training and availability of highly qualified testing personnel. This naturally limited the number of patients who could be tested and precluded the administration of the complete test battery to all patients at both testing time points. This stringent requirement thus restricted the study to a smaller patient group of 48 patients (median age 51.6 years, range 22–85 years, 19 females and 29 males), who provided a complete pre- and post-OP dataset.

### Patient characteristics

In 32 of these patients (66%), surgery was performed in a primary setting, whereas 16 patients (33%) were operated on subsequent to tumor recurrence. The majority of patients (n=37, 77%) were diagnosed with a glioma (IDH-wild-type glioblastoma in 16 patients; IDH-mutated WHO grade 3–4 gliomas in 9 patients; and IDH-mutated WHO grade 2 gliomas in 12 patients). In 8 patients (16%), a cerebral metastasis was diagnosed, and 2 patients presented with other diagnoses.

In 16 patients (33.3%), the tumor was right-sided, whereas in 32 patients (66.6%) it was left-sided. In 18 patients, the tumor was mainly located in the frontal lobe; in 13 patients, in the temporal lobe; in 10 patients, in the parietal lobe; in 3 patients, in the insular lobe; and in 4 patients, in the occipital lobe.

### Test results

As our goal in this paper is to demonstrate the feasibility and efficacy of this test battery, we restrict ourselves to the analysis of pre- and post-OP scores of the 48 patients who completed all perioperative tests. We then present a longitudinal exploration of a single case.

**Table 2** (descriptive statistics) and **Table 3** describe the statistical results. For further information, see the **Supplementary Material**.

**Table 2:** Descriptive statistics (by percentile).

Test type	Time point	Mean % correct (SD)	Min	P25	P50	P75	Max
LING-SPL	Pre-Op	96.15 (6.03)	68.75	93.75	98.13	100.0	100.00
	Post-Op	88.78 (14.43)	53.13	80.63	95.00	100.0	100.00
LING-CPX	Pre-Op	74.83 (21.49)	20.83	62.50	79.17	91.67	100.00
	Post-Op	60.42 (24.33)	12.50	39.58	64.58	79.17	100.00
STD	Pre-Op	94.25 (-6.79)	75.83	92.08	97.08	99.00	100.00
	Post-Op	82.5 (25.75)	3.33	74.92	94.36	98.42	100.00

**Table 3:** Results of permutation and KS tests for differences between pre- and postoperative test types.

A			
Pre-OP			
*X*	*Y*	*mean_diff*	*p value*
LING-SPL	LING-CPX	26.39	1.00E-05
*LING-SPL*	*STD*	*2.88*	*0.09*
LING-CPX	STD	-23.51	1.00E-05
Post-OP			
*X*	*Y*	*mean_diff*	*p value*
LING-SPL	LING-CPX	20.66	1.00E-05
*LING-SPL*	*STD*	*0.74*	*0.29*
LING-CPX	STD	-19.92	1.00E-05
B			
*Time point*	*Comparison*	*KS_a_*	*p*
Pre-Op	LING-CPX vs Standard	2.858	< 0.0001
	LING-CPX vs LING-SPL	3.572	< 0.0001
	LING-SPL vs Standard	1.735	0.0049
Post-Op	LING-CPX vs Standard	2.450	< 0.0001
	LING-CPX vs LING-SPL	2.858	< 0.0001
	*LING-SPL vs Standard*	*1.123*	*0.1607*

Pairwise statistical comparisons of score distributions across the three test types, applied separately for the pre- and postoperative assessments: linguistically simple (LING-SPL), linguistically complex (LING-CPX), and standard (STD) tests (non-significant results are shown in italics).

A. Differences between means detected by a permutation test (105 iterations);

B. Differences in score distributions detected by a Kolmogorov-Smirnov (KS) test, applied separately for pre- and postoperative assessments. KSa - asymptotic KS statistic; P - probability of obtaining a KSa above the reported value. The p-values of all significant results are exceedingly high; correction for multiple comparisons would not alter this pattern.

Here is a bullet-point summary of our results:

- Patients’ performance on standard word-level tests and simple sentence-level tests was high and similar both pre- and postoperatively.
- When tests were grouped by type (complex vs simple), the complex tests were significantly more sensitive than all others (p< 0.0001); no other comparison was significant.
- All individual comparisons between complex and simple app tests (9/9) were significant.
- Individual comparisons between standard and new sentence-level comprehension tests resulted in significantly larger error rates for every complex test, and for most simple tests, in both the pre- and postoperative phases (**Supplementary Table 1**).
- The postoperative scores were slightly lower but largely comparable overall.
- All tests demonstrated an effect of hemisphere postoperatively: performance on both pre- and postoperative tests was significantly worse for patients with left-hemispheric tumors (Standard (STD): p<0.005, SPL: p<0.03, CPX: p<0.003) (data not shown).
- A lobe (frontal vs temporal) x test type (STD vs CPX) interaction was found to be significant in the left hemisphere (p<0.02) (data not shown).
- The cumulative distributions of both the pre- and post-operatively applied complex linguistic tests (**Figure 1**, **Table 3B**) deviate clearly from the other test types, and lean leftward. The Kolmogorov-Smirnov-test confirmed these differences, indicating significant differences between the complex linguistic tests and the other test types, with highlights below (full set in **Table 3B**):
- a. complex-sentence vs. standard test: p < 0.0001,
  b. complex vs. simple-sentence: p<0.0001, both, pre- and post-operatively.
  c. the differences between the simple-sentence and standard tests were smaller (Pre-operatively: p = 0.0049, post-operatively: p = 0.1607, n.s.).
  d. the score distribution of the pre-operatively applied standard tests was slightly wider than the distribution of the simple-linguistic test scores.
- The score distribution of the preoperatively applied STD tests was slightly wider than that of the LING-SPL test scores.

### A longitudinal case study

**Figure 2:**
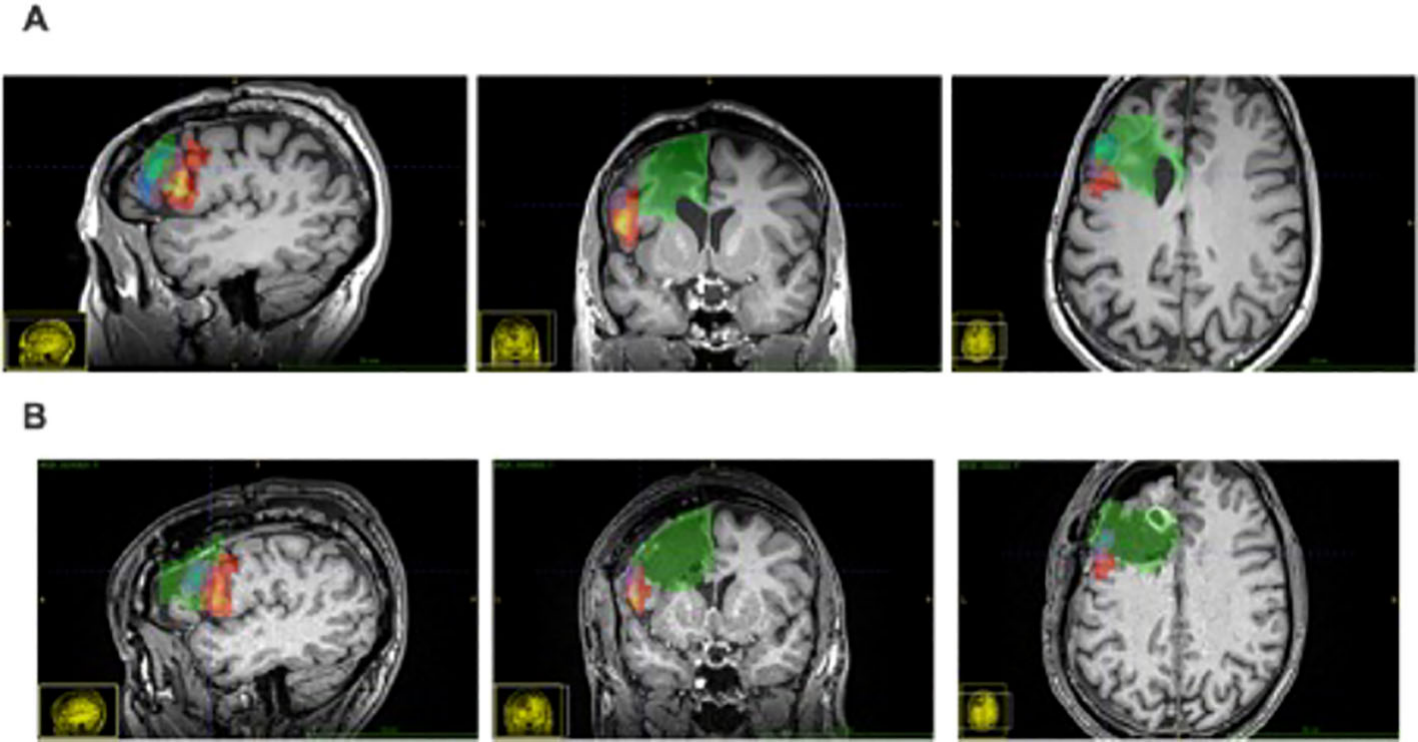
Patient’s MR scans. **(A)** pre-surgical T1 scans, **(B)** post-surgical scans; Green, lesion mask (incomplete anteriorly); Orange, BA 44; Blue, BA 45. Note that the ventricles and sulci appear larger in A than in B, possibly due to post-surgical edema.

**Figure 3:**
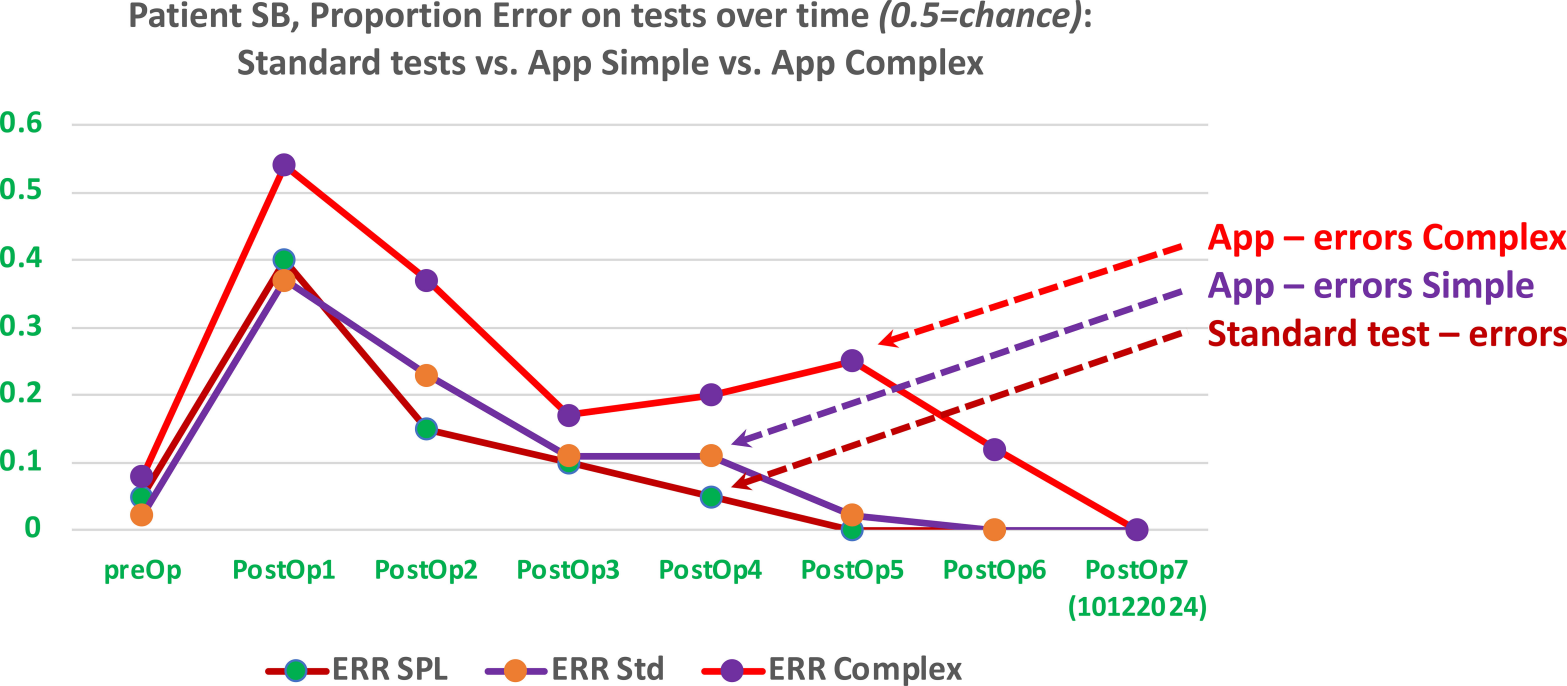
Patient’s performance across seven sequential tests, taken within a 4-month period. Performance on standard tests (ERR Std) compared with the app’s simple and complex tests. The latter are more sensitive, revealing higher levels of comprehension errors in almost all tests.

Patient SB, a 52-year-old patient, was first diagnosed in 2012 with a diffuse astrocytoma, CNS WHO grade 2, located in the left frontal lobe (**Figure 2**). Because of a slow disease progression (increased T2/FLAIR imaging tumor tissue) during the course of therapy, re-resection was discussed in an awake setting. The patient’s postoperative hospital stay was longer than usual, and the patient was retested four months later. This enabled a longitudinal assessment of the patient’s progress across the different tests (**Figure 3**).

In order to determine which cortical areas in the patient’s brain were affected by the tumor and the surgical intervention, the Jülich-Brain Atlas of cytoarchitectonic areas and nuclei, available at EBRAINS (https://www.ebrains.eu/brain-atlases/reference-atlases/), was mapped onto the patient’s brain. First, masks covering the tumor and surgical lesion were manually drawn on FLAIR images of the patient, acquired before and after the surgical intervention (pre- and postoperative scans). These masks were then transformed on T1-weighted MR images acquired at the same time points using a rigid-body co-registration. Next, a non-linear registration of the patient’s T1-weighted MR images to the reference brain of the Jülich-Brain Atlas (29) was calculated, yielding a non-linear transformation between both brains, which was used to transform the Jülich-Brain Atlas into the patient’s brain images. **Figure 2** shows the tumor and lesion mask in green, and the transformed maps of areas 44 and 45 in red-yellow and blue, respectively. The results show that area 44 is affected only to a minor degree, whereas 54% and 90% of area 45 were directly affected pre- and postoperatively, respectively (**Table 4**).

**Table 4:** Patient SB’s calculated extent of lesion (by the Jülich Brain Atlas) to the subparts of Broca’s region.

Area	Pre-surgical % lesioned	Post-surgical % lesioned
45	54.9	89.5
44	1	9.4

## Discussion

Without sentences, communication is extremely limited. Thus, there is little doubt that complex language abilities are critical for the patient’s QoL. Nevertheless, the majority of perioperative language testing in neurooncology does not exceed the word level, so that the reflection of complex language abilities is very poor. Preserving such abilities across all modalities during the resection of gliomas is therefore a high neurosurgical priority. In fact, we saw that the scores of the word-level STD tests are lower than those of the LING-SPL tests, indicating that, like everyone else, patients find simple sentence-level tasks easier than word-level tasks, suggesting that sentences are the most natural means of human communication. Importantly, the LING-CPX sentences, which were the most sensitive, feature types that are used by everyone on a daily basis. Their importance for QoL is thus very high.

Here, we presented preliminary results of a new pre- and postoperative battery of language tests that allow for resolution that is demonstrably higher than that of the tests that are commonly used. Sentence complexity (CPX vs. SPL) emerged as the factor that was most sensitive to the presence of a tumor and that is most profoundly affected by the surgical process. Indeed, the comprehensive statistical comparison with standardly used tests strongly suggests that this new set should become part of the standard toolkit that oncology neurosurgeons use for the evaluation of language abilities. Our tests are, moreover, rather robust: although our results are based on a small number of patients with different tumor localizations and different tumor entities, this noisy dataset still manifested significant effects, including a right-left hemispheric difference: pre- and postoperative success rates were lower when the tumor was located on the left side, especially if the tumor was located in the frontal lobe. This is consistent with known neuropsychological and neuroimaging results (cited in the introduction). Our results thus underscore the urgent need to incorporate tests like this into the intraoperative awake test battery and to extend them to other modalities (reading, writing, production, and repetition). We will soon report the results of our ongoing study of intraoperative language mapping with this new tool.

Some recent papers on awake neurosurgery have presented a broad range of intraoperative language tests (30) aimed at localizing language functions, some of which probed aspects of sentence-level (19, 20, 31, 32). None, however, contained a systematic, evidence-based test battery, nor has a comparative evaluation of different test types been published that compares the sensitivity and efficacy of the tests in use. Our study is the first to offer both.

In addition, we note that most sentence-level investigations in other relevant studies have been restricted to the Token Test (33). Developed in the early 1960s, this test is not structured systematically along linguistic lines and is certainly not informed by what is currently known about how language is cerebrally represented. Its intraoperative localizing value is, therefore, rather limited, as our results clearly indicate. Lastly, the Token Test’s scoring system is not structured to distinguish between sentence types. Our test battery answers these concerns and, as we showed, is demonstrably better at detecting comprehension deficits, as evident from direct comparisons between patients’ scores on our tests and those on the Token Test.

Our test battery, therefore, seems to fill a much needed lacuna in testing–sentence-level language assessment, especially in comprehension—and should be incorporated into standard test batteries administered both outside and inside the OR. As such, they must be part of language testing pre-, intra-, and postoperatively.

### Study limitations

We present perioperative monocentric data from a relatively small patient cohort, which is also heterogeneous with respect to age, lesion site, handedness, tumor size, and tumor type. The small cohort size is a result of the test battery size. In addition to the need for testing it in multiple centers, our preliminary results require further elucidation and intraoperative validation. We have begun producing and analyzing such results, and we seek to extend them in the near future. Finally, extending our battery to language modalities beyond comprehension is also needed. We hope that this will soon become possible.

## Conclusions

In sum, our design comprises several well-balanced, high-resolution sentence-level tests, which focus on sentences and systematically manipulate their structural complexity in a manner that is said to expose a core aspect of human communicative ability. We recommend its use in glioma resection perioperatively and intraoperatively to avoid permanent language deficits.

## Data Availability

The original contributions presented in the study are included in the article/**Supplementary Material**. Further inquiries can be directed to the corresponding author.
