# Supplementary material for: A novel high-resolution test battery for language mapping in awake craniotomy: preliminary perioperative results in neurooncologic patients
Source: Front Oncol. 2026 May 18;16:1762147. doi: 10.3389/fonc.2026.1762147 (PMC13222778; doi:10.3389/fonc.2026.1762147)
Supplement: Supplementary file 1 [file Table1.docx]

**Supplemental Table 1**

Significant individual permutation tests of patients’ (n=48) pre-operative scores on all tests: 11 Standard tests (Word ID, Counting, Weekdays, Months, Picture naming, Famous faces, STROOP, Reading, Calculation, TOKEN, PPTT), 3 Simple (SPL) and 3 Complex (CPX) app tests (Questions=Q, Requests=REQ, Comparisons=COMP). Note the high p-values, leading to the conclusion that over half of these will survive correction for multiple comparisons.
